# High Manganese Tolerance and Biooxidation Ability of *Serratia marcescens* Isolated from Manganese Mine Water in Minas Gerais, Brazil

**DOI:** 10.3389/fmicb.2017.01946

**Published:** 2017-10-09

**Authors:** Natália R. Barboza, Mônica M. C. A. Morais, Pollyana S. Queiroz, Soraya S. Amorim, Renata Guerra-Sá, Versiane A. Leão

**Affiliations:** ^1^Laboratório de Bioquímica e Biologia Molecular, Departamento de Ciências Biológicas, Instituto de Ciências Exatas e Biológica (NUPEB), Universidade Federal de Ouro Preto, Ouro Preto, Brazil; ^2^Unileste, Ipatinga, Brazil; ^3^Laboratório de Bio&Hidrometalurgia, Departamento de Engenharia Metalúrgica e de Materiais, Escola de Minas, Universidade Federal de Ouro Preto, Ouro Preto, Brazil

**Keywords:** manganese, bioremediation, *Serratia marcescens*, manganese oxidation, biooxidation

## Abstract

Manganese is an important metal for the maintenance of several biological functions, but it can be toxic in high concentrations. One of the main forms of human exposure to metals, such as manganese (Mn), is the consumption of solar salt contaminated. Mn-tolerant bacteria could be used to decrease the concentration of this metal from contaminated sites through safer environmental-friendly alternative technology in the future. Therefore, this study was undertaken to isolate and identify Mn resistant bacteria from water samples collected from a Mn mine in the Iron Quadrangle region (Minas Gerais, Brazil). Two bacterial isolates were identified as *Serratia marcescens* based on morphological, biochemical, 16S rDNA gene sequencing and phylogeny analysis. Maximum resistance of the selected isolates against increasing concentrations of Mn(II), up to 1200 mg L^-1^ was determined in solid media. A batch assay was developed to analyze and quantify the Mn removal capacities of the isolates. Biological Mn removal capacities of over 55% were detected for both isolates. Whereas that mechanism like biosorption, precipitation and oxidation could be explaining the Mn removal, we seek to give an insight into some of the molecular mechanisms adopted by *S. marcescens* isolates. For this purpose, the following approaches were adopted: leucoberbelin blue I assay, Mn(II) oxidation by cell-free filtrate and electron microscopy and energy-dispersive X-ray spectroscopy analyses. Overall, these results indicate that *S. marcescens* promotes Mn removal in an indirect mechanism by the formation of Mn oxides precipitates around the cells, which should be further explored for potential biotechnological applications for water recycling both in hydrometallurgical and mineral processing operations.

## Introduction

Contaminated mine water remains a major problem worldwide and is associated with severe environmental, socio-economic, and public health impacts. It is mostly characterized by extreme pH (acidity or alkalinity), high salinity levels, particularly sulfate, Al, sundry toxic metals such as Fe, Cd, Co, Cu, Mo, Zn, Ni, and V, and sometimes even radionuclides (Imtiaz et al., 2015; Beane et al., 2016; Sethurajan et al., 2016). In Brazil and the state of Minas Gerais in particular, mining activities have a long history and have played a major role in both economic development and environmental pollution throughout the country (Instituto Brasileiro de Mineração [IBRAM], 2012; Massante, 2015). Although significant progress has recently been made to address mine water management, environmental pollution due to the disposal of untreated mine water remains a major problem worldwide (Klein et al., 2014). In the specific case of manganese (Mn), several lines of evidence suggest a positive association between environmental exposures, which are common and cumulative in a lifetime, and development of neurodegenerative diseases. Thus, environmental or occupational exposure to Mn has been implicated in neurodegeneration related to impaired dopaminergic (DAergic), glutamatergic and GABAergic transmission, mitochondrial dysfunction, oxidative stress, and neuroinflammation (Peres et al., 2016).

Normally Mn is removed by adding some basic chemical to the drainage prior to returning them to the environment. Chemical oxidation could be performed to Mn removal by adding strong oxidizing agents (e.g., potassium permanganate, hypochlorite or ozone) or by aeration (Silva et al., 2010), although they are often expensive and inefficient and produce secondary pollutants such as toxic byproducts (Ehrlich, 1999; Das et al., 2011).

As an alternative, bioremediation by Mn(II)-oxidizing bacteria has been shown to be a viable strategy for metal removal (Gallard and van Gunten, 2002; Pacini et al., 2005; Mariner et al., 2008; Luan et al., 2012). Biological Mn oxidation can occur by two mechanisms. The first mechanism is the direct mechanism, which is mediated by cellular components such as proteins. This mechanism has been studied for several years and the role of MCO enzymes in the oxidation of Mn(II) by several species of bacteria has been demonstrated (Brouwers et al., 1999, 2000; Tebo et al., 2004, 2005; Dick et al., 2008; Mayhew et al., 2008; Soldatova et al., 2012; Geszvain et al., 2013; Su et al., 2013). The second mechanism for biological Mn oxidation is the indirect mechanism, which occurs when the metabolism or growth of microorganisms changes the pH or the redox conditions of the environment or releases metabolic products that can chemically oxidize Mn(II) to Mn(III) or Mn(IV) (Nealson et al., 1988; Tebo et al., 2004; Learman et al., 2011). Richardson et al. (1988) reported that cyanobacteria and algae could promote Mn oxidation by an indirect mechanism. In both cases, Mn(II) oxidation occurred as a result of environmental modifications of pH and redox potential (Eh). Hullo et al. (2001) also demonstrated a non-enzymatic Mn(II) oxidation mechanism mediated by *Bacillus subtilis*. They observed that the oxidation occurred due to the increased pH promoted by *B. subtilis.* Although these bacteria contain a spore coat protein, CotA, that is similar to laccases, this protein did not play any role in the Mn(II) oxidation.

Many bacterial strains that are capable of promoting the oxidation of Mn(II) to Mn(IV) by indirect, indirect, or both mechanisms have been identified. *Bacillus* sp. SG-1, *Pseudomonas putida* strains MnB1 and GB-1, and *Leptothrix discophora* strains SS-1 and SP-6, are examples of bacteria that have been extensively studied for bioremediation (Adams and Ghiorse, 1987; van Waasbergen et al., 1996; Hope and Bott, 2004; Tebo et al., 2005; Geszvain et al., 2013). Johnson and Hallberg (2003), Tuffin et al. (2006), and Wang et al. (2011) reported that environmental characteristics such as extreme conditions (e.g., pH, metal concentration, etc.) influence the microbial community composition.

In a previous report, we showed that isolates belonging to the genera *Stenotrophomonas*, *Bacillus*, and *Lysinibacillus* from water samples collected from a Mn mine in the Iron Quadrangle region (Minas Gerais, Brazil) were able to perform Mn(II) oxidation by a non-enzymatic pathway (Barboza et al., 2015), and the isolates used in this present article were also isolated from the same place. *Serratia marcescens* shows promise for the development of biotechnological and bioremediation processes, for example, in the decolorization of synthetic dyes (Verma and Madamwar, 2003) and the industrial effluent known as black liquor, and the removal of organophosphorus pesticides from soils (Cycon et al., 2013). Although the role of *S. marcescens* in iron and Mn oxide formation during pipe corrosion has been investigated (Rajasekar et al., 2007a,b), the potential for Mn(II) tolerance and removal is not understood. Thus, in this work, we seek to investigate the Mn(II) tolerance and oxidation capacity of *S. marcescens* isolates with the goal of identifying new isolates with biotechnological potential for Mn removal from mine waters.

## Materials and Methods

### Sample Collection and Isolation of Mn-Tolerant Strains

Several samples were obtained from Mn mine water collected from the Iron Quadrangle region (Minas Gerais, Brazil). To select Mn(II)-tolerant strains, the samples were appropriately diluted and spread on agar plates with K medium (0.001 g L^-1^ FeSO_4_⋅7H_2_O; 2 g L^-1^ peptone, 0.5 g L^-1^ yeast extract, and 10 mM HEPES buffer, pH 7.5) supplemented with 50 mg L^-1^ Mn(II) as MnSO_4_⋅H_2_O. After 7 days of incubation at 28 ± 2°C, two colonies growing on the plates were isolated and selected for the subsequent assays.

### Evaluation of Mn(II) Tolerance

The isolated bacteria were spread on solid K medium supplemented with various Mn(II) concentrations (140–1200 mg L^-1^) to determine their maximum tolerance to this metal. The MTC was defined as the highest concentration of the contaminant for which bacterial growth could be observed after 7 days of incubation at 28 ± 2°C. Two strains isolated from the sediments, named CL11 and CL35 pending their subsequent identification, with the capability to tolerate high Mn(II) concentrations, were selected, characterized, and identified for further Mn(II) removal studies.

### Characterization and Identification of CL11 and CL35 Isolates

The isolates were characterized based on their morphology, Gram staining, and oxidase, catalase, and biochemical tests. The metabolic profiles were assessed using the Bactray system (LaborClin, Paraná, Brazil) following the manufacturer’s protocol. Subsequently, the results of the biochemical tests were analyzed using the Bactray software. This program utilizes a dataset of the metabolic profiles of many bacteria and compares the experimental results with the dataset.

Identification of the CL11 and CL35 strains through molecular methods was also carried out. For this, the 16S rRNA gene was amplified and sequenced using forward 27F and reverse 1942R primers (Yang et al., 2013). For gDNA extraction, the Wizard Genomic kit (Promega) was used following the manufacturer’s recommendations and samples were stored at 4°C until use. Prior to gDNA extraction, the isolates were grown in K medium without Mn(II) (0.001 g L^-1^ FeSO_4_⋅7H_2_O, 2 g L^-1^ peptone, 0.5 g L^-1^ yeast extract, and 10 mM HEPES buffer at pH 7.5) overnight at 30°C under constant stirring at 150 rpm. The cells were recovered via centrifugation at 14,681 × *g* for 5 min and used for gDNA extraction.

PCR amplification was performed in a reaction mixture consisting of Taq buffer 1X, 1.5 mM MgCl_2_, 0.2 mM of each deoxynucleotide, 0.2 mM of each primer, 2.5 U Taq DNA polymerase (Thermo Scientific *Taq*DNA Polymerase, Fermentas), 1 ng DNA template, and water to bring the total volume to 25 μL. A thermocycling program was carried out using the following protocol: an initial denaturation step (94°C, 5 min), followed by 35 cycles of denaturation (94°C, 45 s), annealing (63°C, 1 min), and extension (72°C, 2 min). A single final extension step (72°C, 20 min) concluded the reaction (Barboza et al., 2015). Purification of the PCR products was carried out as described previously (SambrooK et al., 1989).

The amplicons were sequenced in the forward direction using a BigDye Terminator kit (Applied Biosystems) according to the manufacturer’s instructions and analyzed using an automated DNA sequencer (3500 Genetic Analyzer, Applied Biosystems). Triplicates were used to construct the consensus sequences using the ClustalW tool (Aiyar, 2000). The bacterial sequence was used to produce phylogenetic trees constructed using the neighbor-joining method and the Jones-Taylor-Thornton model (Saitou and Nei, 1987) using the consensus sequences. Bootstrap resampling analysis of 1,000 replicates was performed to estimate the confidence levels of the tree topologies and the FigTree 1.4 software was used for phylogenetic analyses. The partial sequences of the 16S rRNA gene sequences of the *S. marcescens* isolates were deposited in GenBank under the accession numbers KX444553 (CL11) and KX44455 (CL35).

### Mn Removal Assay

Manganese removal experiments were carried out with synthetic solutions of fresh K medium supplemented with ca. 45 mg L^-1^ Mn(II). The isolate stocks stored at -80°C were grown in 100 mL of K medium without Mn(II) at 30°C under constant stirring (150 rpm) for 24 h. Then, this culture was transferred to 250-mL flasks containing 90 mL of fresh K medium without Mn(II). Subsequently, 10 mL of the culture (with an approximate optical density of 1.0 at 600 nm) was transferred to 90 mL of fresh K medium with ca. 45 mg L^-1^ Mn(II), and the flasks were incubated at 30°C under constant stirring (150 rpm) for 7 days. Samples were collected periodically to measure the Mn(II) concentration (ICP-OES, Varian 725), pH, and bacterial growth (via the optical density at 600 nm using a Hitachi 2800 A series spectrophotometer). The Mn quantification was enhanced as previously described by Barboza et al. (2015): an aliquot of 4 ml of each sample was centrifuged for 15 min at 14,681 × *g* and then filtered through a 0.22-μm membrane. The filtrate was diluted ten times in distilled water, and acidified with HCl (1:1) solution. Mn removal from the culture medium was measured by the decay of Mn concentration in the samples, via ICP-OES assay. Briefly, the ICP-OES analyzes were performed using the following parameters: accuracy of 5%, detection limit of 0.001 mg L^-1^ and limit of quantification of 0.01 mg L^-1^. For the equipment calibration, after the initial adjustments, which comprise the optical stabilization and calibration of the spectral lines (Mn wavelength: 257.610 nm), the analytical calibration curve was constructed which comprises the points of the curve (0; 2.5; 5.0; and 10 mg L^-1^ of Mn). Then, the external standard was analyzed (5 mg L^-1^ of Mn) and subsequently the analysis of the samples was started. All measurements were made in triplicate and the ICP-OES was configured to report the average value, respecting the accuracy of the 5% method. Control flasks (abiotic experiments) with the pH adjusted to 7.5, 8.0, or 8.2 were maintained under similar conditions, and bacterial growth was prevented by adding Nipagin (0.14%)/Nipazol (0.1%). The experiments were performed in triplicate. Results were compared using the ANOVA (ONE-WAY) (Turkey test) and were considered statistically significant at *p* < 0.0001. The software used was GraphPad Prism.

### Mn(II) Oxidation by Cell-Free Filtrate

The cell-free filtrates were prepared as described previously (Learman et al., 2011). The cell-free filtrate The cell free filtrate of each sample was divided into two equal parts, one of which was treated with proteinase K (100 μg mL^-1^, Promega) at 37°C for 3 h before adding of, approximately 45 mg L^-1^ of Mn(II). The flasks (i.e., with or without proteinase K) were incubated at 37°C and 150 rpm for 7 days. Samples were collected periodically to evaluate the Mn removal and Mn oxidation, by ICP-OES and leucoberbelin blue I dye (LBB, Sigma–Aldrich, United States) assay, respectively.

### Mn(II) Oxidation Assays

To assess whether Mn removal occurred via Mn oxidation, 0.1 mL samples of the cultured CL11 and CL35 isolates grown with ca. 45 mg L^-1^ Mn(II) for a week were mixed with 0.5 mL of 0.04% LBB in 45 mM acetic acid (Krumbein and Altmann, 1973). As negative controls, K medium with or without 45 mg L^-1^ Mn(II) or with only the isolate were mixed with LBB and a Mn carbonate (MnCO_3_) solution. As a positive control, K medium with Mn oxide (MnO_2_) was used.

### Electron Microscopy and Energy-Dispersive X-Ray Spectroscopy (EDX) Analyses

The CL11 and CL35 isolates cultured in liquid K medium with ca. 45 mg L^-1^ Mn(II) for 7 days, as previously described, were analyzed via TEM and SEM. SEM/EDX assays were carried out using an FEI Quanta 200 FEG, and TEM/EDX assays were performed using a Tecnai G2 12 Spirit Biotwin FEI-120 kV and a Tecnai G2 20 SuperTwin FEI-200 kV. Electron microscopy were carried out in the Center of Microscopy at the Universidade Federal de Minas Gerais, Belo Horizonte, MG, Brazil.

## Results

### Phenotypic and Phylogenetic Analysis of the Isolates

**FIGURE 1:**
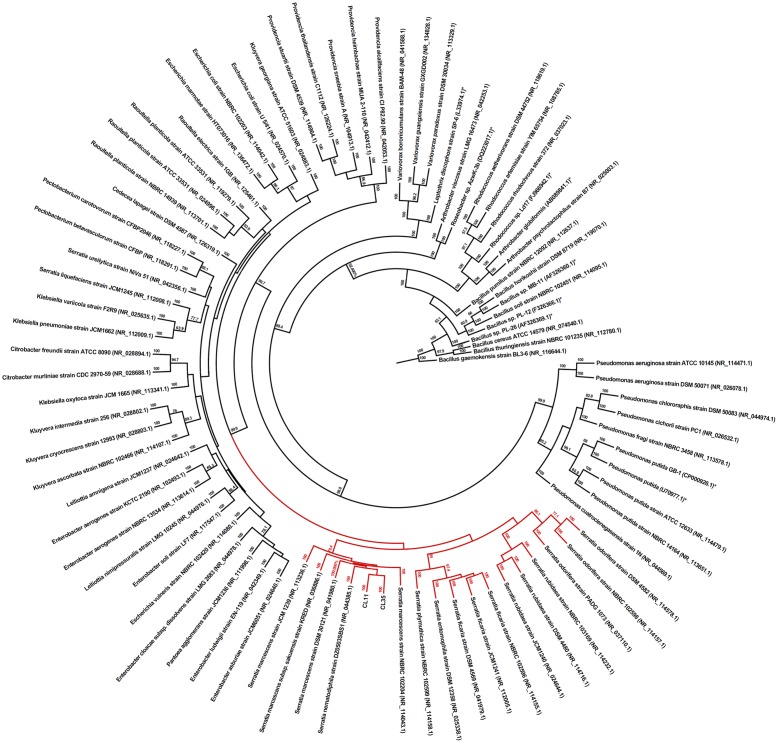
Phylogenetic identification of the CL11 and CL35 isolates. The relationships between the 16S rRNA gene sequences of the isolates and the closest GenBank sequences with the 16S rRNA gene from previously reported Mn(II)-oxidizing bacterial strains (labeled with ^∗^) are shown. The GenBank accession numbers of the sequences are shown in brackets. Bootstrap values of ≥50% with 1,000 replicates are indicated at the branch points.

Both of the isolates were rod-shaped, non-pigmented, Gram-negative, and catalase and cytochrome oxidase negative (**Table 1**). The biochemical test results were analyzed using the Bactray software, and both isolates were identified as *S. marcescens* with a confidence level of 99.97%. To confirm this identification, we also used a molecular approach for phylogenetic identification. The 16S rRNA genes from both isolates were amplified, sequenced, and found to exhibit 91.5 and 81.17% of 16S RNA coverage compared with the *S. marcescens* sequences deposited in the database for CL11 (accession number KX444553) and CL35 (accession number KX444554), respectively. The BLAST (Altschul et al., 1990) was used to search for similar sequences from GenBank. Furthermore, the 16S rRNA sequences of several well-known Mn(II)-oxidizing bacteria were included in the phylogenetic study. We found that the isolates CL11 and CL35 were closely clustered with the genus *Serratia*, primarily with *S. marcescens* (**Figure 1**). Because the phenotypic and phylogenetic characterizations showed the same results, we identified both isolates as *S. marcescens.*

**Table 1:** Phenotypic profiles of the isolates CL11 and CL35.

	Results	
	
Conducted test	CL11	CL35
Colony form	Rod	Rod
Gram staining	-	-
Catalase	+	+
Cytochrome oxidase	-	-
*ortho*-Nitrophenyl galactoside	-	-
Arginine decarboxylase	-	-
Lysine decarboxylase	+	+
Ornithine decarboxylase	+	+
H_2_S	-	-
Urease	+	+
Voges–Proskauer	+	+
L-Phenylalanine	-	-
Indole	-	-
Citrate	+	+
Malonate	-	-
Rhamnose	-	-
Adonitol	+	+
Salicin	+	+
Arabinose	-	-
Inositol	+	+
Sorbitol	-	-
Sucrose	+	+
Mannitol	+	+
Raffinose	-	-


*Symbols: positive (+); negative (-).*

### Evaluation of Mn(II) Tolerance

**FIGURE 2:**
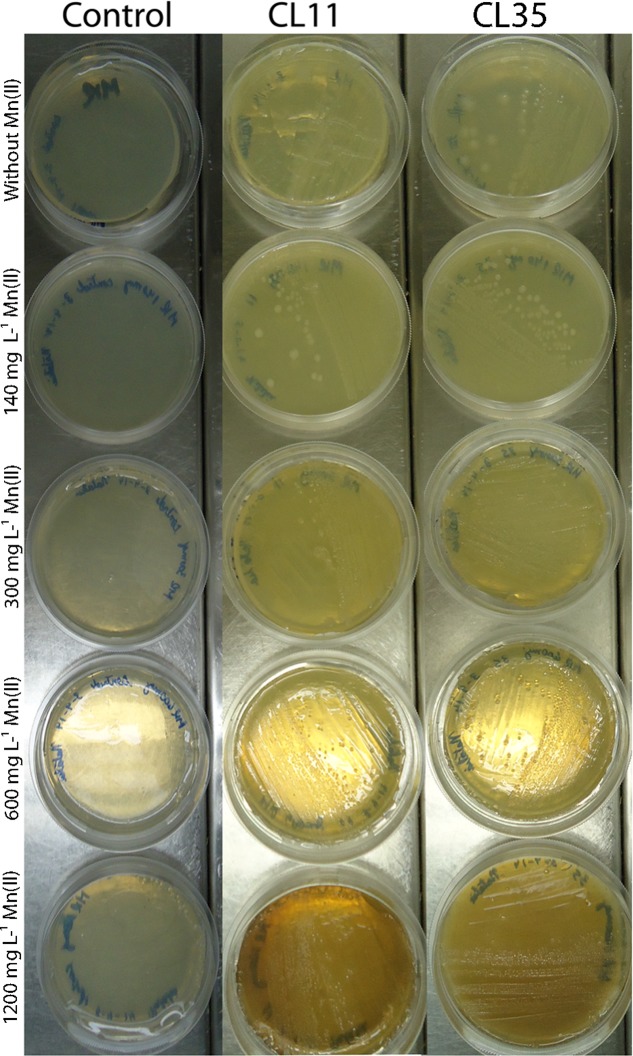
Mn(II) ion tolerance. Isolates CL11 and CL35 were grown on K medium supplemented with various concentrations of Mn(II) ions. The isolates were cultured at 30°C for 2 weeks.

Initially, the isolates were grown in solid K medium containing different Mn(II) ion concentrations (140, 300, 600, and 1200 mg L^-1^). The capability for Mn(II) removal was verified by the development of brown color in the colonies or in culture medium. As shown in **Figure 2**, both the Cl11 isolate and the CL35 isolate were able to grow at the Mn concentrations used.

### *S. marcescens* Promotes Mn Removal by Mn(II) Oxidation

**FIGURE 3:**
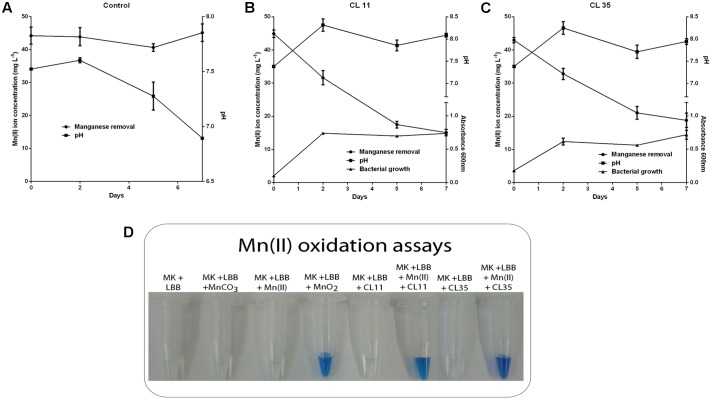
Decay of manganese concentration during manganese removal assay by the CL11 and CL35 isolates and qualitative determination of Mn(II) oxidation. The time course plots for Mn(II) removal, pH changes, and cell growth (where applicable) for **(A)** the control experiments and **(B)** the CL11 and **(C)** CL35 isolates. The cells were grown in K medium for 1 week. **(D)** For the qualitative determination of Mn(II) oxidation, 0.04% of LBB reagent was added to the strains grown in K medium with 45 mg L^-1^ Mn(II) for 1 week. The error bars indicates the standard deviation of the biological triplicate.

To investigate the further use of the wild *S. marcescens* isolates in bioremediation approaches, batch Mn removal experiments were performed. The ability to Mn removal of the isolates was tested with respect to incubation time (0 min to 7 days) as well as the initial ca. 45 mg L^-1^ Mn(II) concentration and pH conditions. We found that isolates CL11 and CL35 were able to remove 56.37–66.42% of the Mn(II) from a synthetic solution containing approximately 45 mg L^-1^ Mn(II) after 1 week. We also observed an increase in pH (7.38 to 8.0; **Table 2** and **Figures 3B,C**). In the abiotic control experiment, no Mn removal was observed from the culture medium, and the pH decreased from 7.52 to 6.89.

**Table 2:** Mn(II) ion removal by the CL11 and CL35 isolates and pH variation during small-scale batch experiments over a 1-week period.

	Mn(II)	Initial Mn(II)	Residual Mn(II)	Initial	Final
	removal	ion concentration	ion concentration	pH	pH
Control	0%	44.23 mg L^-1^	45.13 mg L^-1^	7.52	6.89
CL11	66.42%^∗#^	44.89 mg L^-1^	15.07 mg L^-1^	7.38	8.08
CL35	56.37%^∗^	42.99 mg L^-1^	18.74 mg L^-1^	7.38	7.94
pH 7.5	7.05%	40.82 mg L^-1^	37.93 mg L^-1^	7.54	7.43
pH 8.0	50.86%	42.64 mg L^-1^	20.95 mg L^-1^	8.03	8.04
pH 8.2	48.77%	38.75 mg L^-1^	19.85 mg L^-1^	8.23	8.16


*^∗^Statistically different from control and pH 7.5. ^#^Statistically different from pH 8.0 and pH 8.2.*

**FIGURE 4:**
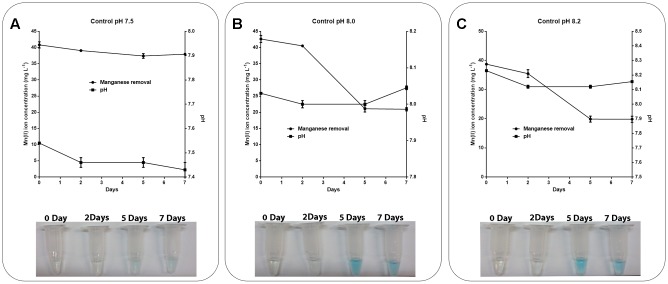
Role of pH in manganese oxidation during manganese removal assay. K medium supplemented with 45 mg L^-1^ Mn(II) at pH values of **(A)** 7.5, **(B)** 8.0, or **(C)** 8.2 was maintained at 30°C under constant stirring (150 rpm) for 7 days. The samples were collected and manganese removal was determined by inductively coupled plasma optical emission spectrometry. Manganese oxidation was detected by the addition of 0.04% LBB reagent to the samples. The error bars indicates the standard deviation of the technical triplicate.

As the pH in the abiotic experiments decreased to 6.89 and the pH of the media containing the isolates increased to approximately 8.0, abiotic experiments were performed using K medium with the pH adjusted to 7.5, 8.0, or 8.2 (**Figure 4**). It was observed that at pH 8.0 or above, the Mn removal reached 50% efficiency (**Figures 4B,C**) and the pH remained constant during the experiments (**Figure 4**). The addition of the LBB reagent in samples collected periodically also demonstrated Mn oxidation at pH 8.0 or above (**Figure 4**).

**FIGURE 5:**
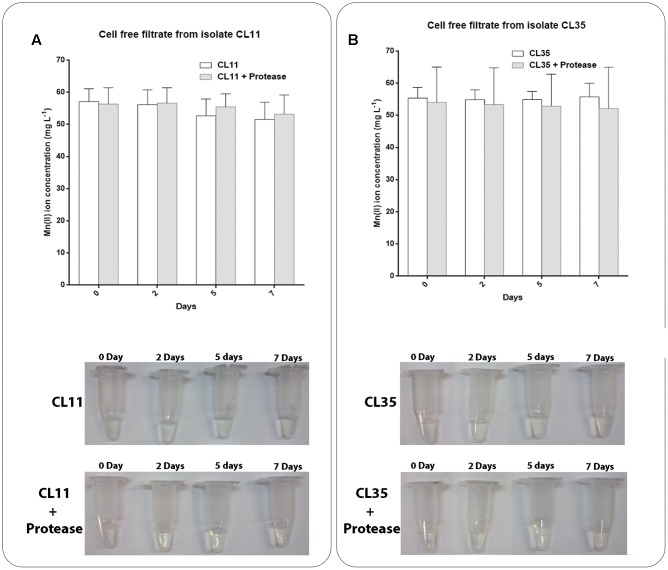
Decay of manganese concentration during manganese removal assay and Mn(II) oxidation in cell-free filtrates of **(A)** CL11 and **(B)** CL35 under standard conditions or after the addition of proteinase K. The isolates were grown in K medium and the supernatant was recovered to evaluate the oxidation of Mn as described in section “Materials and Methods.” Samples were collected periodically and manganese removal was determined by ICP-OES. The presence of manganese oxide was monitored by the addition of LBB to the samples. The error bars indicates the standard deviation of the biological triplicate.

Mn(II) oxidation assays were also performed using cell-free filtrates in either the presence or absence of proteinase K to assess whether any extracellular proteins were responsible for the Mn oxidation. We did not observe Mn removal or oxidation by the cell-free filtrates from the isolates CL11 (**Figure 5A**) or CL35 (**Figure 5B**) in either the presence or absence of the protease. These results indicate that no extracellular proteins were involved in the Mn oxidation by the isolates studied.

### SEM/EDX and TEM/EDX Analyses

**FIGURE 6:**
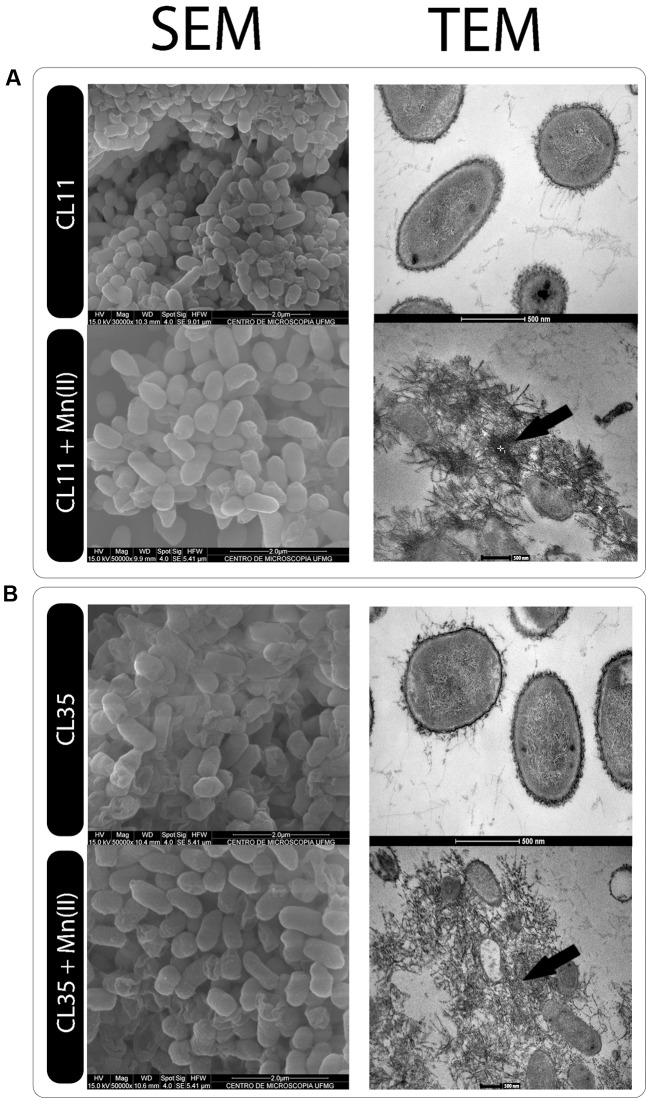
Electron microscopy of the **(A)** CL11 and **(B)** CL35 isolates. SEM and TEM images of the isolates cultured in K medium in the presence or absence of 45 mg L^-1^ Mn(II) for 7 days.

**FIGURE 7:**
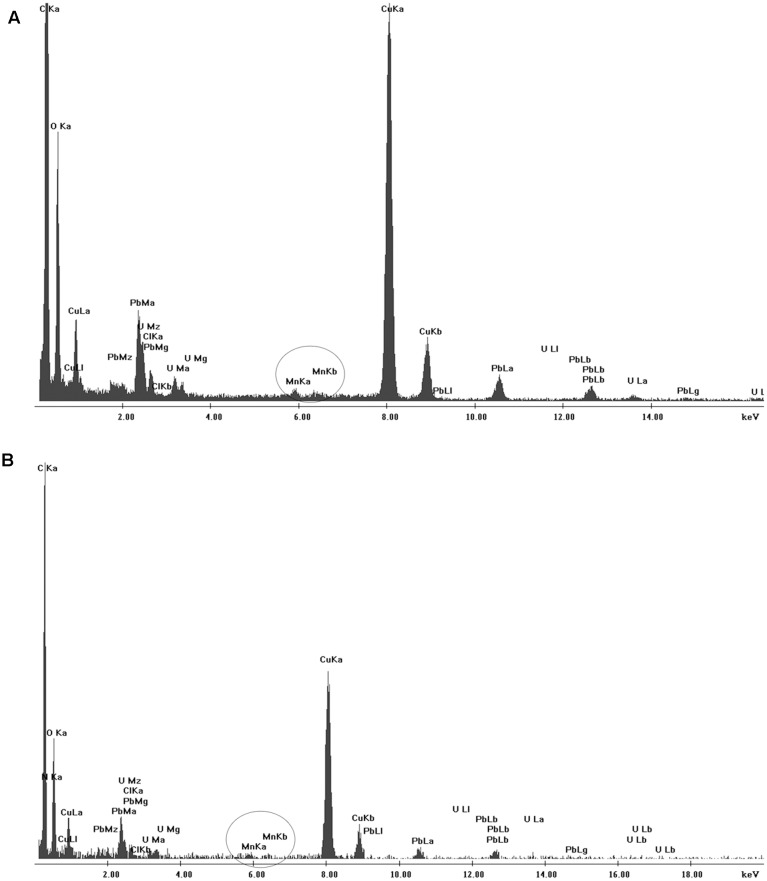
Energy-dispersive X-ray spectroscopy (EDX) results for **(A)** CL11 and **(B)** CL35. The corresponding TEM images are shown in **Figure 6**.

Scanning electron microscopy and TEM analyses revealed no aggregates either on cell membrane of the isolates or within the cells after 7 days of culture (**Figure 6**). However, when the isolates were grown in the presence of Mn(II) ions, we observed the extracellular precipitation of a Mn-containing mineral phase, as revealed by the EDX spectra (**Figure 6**, indicated by arrows, and **Figure 7**).

## Discussion

The Mn could be found in several oxidation states depending on the redox conditions of the environment and the bacteria play an important role in the Mn geochemical cycle. *L. discophora* (strains SS1 and SP-6), *P. putida*, and *Bacillus* sp. SG-1 are the most studied species related to Mn(II) oxidation (Barboza et al., 2016). Similarly, *S. marcescens* isolates were found as part of the microbial community at a Mn mine in the Iron Quadrangle region (Minas Gerais, Brazil) (Barboza et al., 2015).

In this study, we have shown that these isolates are capable of promoting Mn oxidation, at least under laboratory conditions, and that this oxidation probably does not involve extracellular enzymes and is instead due to interaction with a metabolic product from bacteria (e.g., hydroxy acids) or a bacterial cell component.

The role of *S. marcescens* in bioprocesses such as the decolorization of synthetic dyes (Verma and Madamwar, 2003) and the industrial effluent known as black liquor (Chandra et al., 2011) and the bioremediation of environments contaminated with pesticides or uranium (Abo-Amer, 2011; Kumar et al., 2011), was described, however, this is the first study showing the potential of *S. marcescens* in Mn bioremediation processes. Both of the isolates used in this study (CL11 and CL35) were able to grow at Mn(II) concentrations of up to 1200 mg L^-1^ (**Figure 2**) and remove more than 55% of Mn(II) from the culture medium in 7 days (**Table 2** and **Figures 3B,C**). Several studies have shown that different bacteria species tolerate and are able to remove Mn, however, in these work smaller Mn concentrations were used to evaluate the tolerance of bacteria against this metal (Xuezheng et al., 2008; Joshi and Modi, 2013; Barboza et al., 2016; Tang et al., 2016). Despite this, our research group identified other bacteria species, such as *Bacillus* sp., *Stenotrophomonas* sp., and *Lysinibacillus* sp. isolated from the same mine where the strains CL11 and CL35 were obtained, suggesting that the ambient may have influence on the high Mn tolerances of these microorganisms (Barboza et al., 2015).

To investigate which mechanism was used by the isolates CL11 and CL35 to promote Mn removal, control experiments were performed at pH 7.5, 8.0, and 8.2, since a pH value of approximately 8.0 was reached in the Mn-removal experiments with the isolates. It was observed that at pH 8.0 and 8.2, the Mn was removed by oxidation (**Figures 4B,C**) with an efficiency of 50% (**Table 2**). Despite many attempts to buffer the culture medium with HEPES, a pH increase was also observed during Mn removal by the isolates (**Table 2** and **Figures 3B,C**), whereas both negligible Mn removal and no increase in the pH were observed in the abiotic experiments at pH 7.5 or below (**Figures 3A**, **4A**). Therefore, we suggested that bacterial growth induces changes in the pH and favors the chemical removal of Mn(II). Several published finding reinforce that pH is one of the major factors affecting manganese oxidation (Bamforth et al., 2006; Burger et al., 2008; Divekar, 2010; Silva et al., 2010, 2012). Under our conditions, the Mn oxidation was confirmed by addition of the LBB reagent (**Figure 3D**).

We also tested if *S. marcescens*, under the growth conditions used in this work, would be capable of inducing the secretion of proteins that alter the environmental conditions and thus favor the oxidation of the Mn(II) to Mn(III) or Mn(IV). As the Mn removal rate was not affected by proteinase K treatment of the cell-free filtrate, we infer that the Mn removal/oxidation was not dependent on the presence of a protein in the culture medium (**Figure 5**). Together, these results reinforce the hypothesis that the Mn removal mediated by the *S. marcescens* isolates mainly occurs via a mechanism that does not involve extracellular enzymes, although we cannot exclude the participation of intracellular proteins.

Electron microscopy did not reveal any Mn precipitates on the cell membrane or inside the cells, but EDX scanning revealed Mn-containing precipitates around the cells produced by both isolates only in the presence of Mn(II) ions (**Figure 6**, indicated by the arrow). It is known that typically, biogenic Mn oxides are poorly crystalline and Mn oxides are deposited around bacterial cells. For instance, Mn oxides were found to be deposited around the exosporium of *Bacillus* sp. SG-1 (Nealson et al., 1988; Dick et al., 2008; Soldatova et al., 2012), whereas the oxides were found around the sheaths in *L. discophora* (Emerson and Ghiorse, 1992). Miyata et al. (2007) observed similar structures in ultrathin sections of Mn microconcretions, which we also observed using SEM and TEM microscopy.

## Conclusion

Concern for Mn(II) contamination has assumed great importance as it poses potential hazards to the environment, wildlife, and human health. In this study, we confirmed the presence of two Mn-tolerant *S. marcescens* isolates in mine water. The CL11 and CL35 isolates were found to be tolerant to high concentrations of Mn(II) and possess good Mn(II) oxidation capacities. This ability of the *S. marcescens* isolates makes them suitable candidates for the treatment of water contaminated with Mn(II). However, further studies, such as into their interactions with the water environment, are still needed before the application of these strains in field-scale biooxidation.

## Author Contributions

NB carried out the experiments into manganese removal, biooxidation, molecular characterization of the strains, and SEM/EDX and TEM/EDX analyses. NB, RG-S, and VL designed the research and wrote the manuscript. MM, SA, and PQ performed the isolation and biochemical characterization of the isolates. All authors participated effectively in the analyzes and discussions of the results as well as in the writing of the article.

## Conflict of Interest Statement

The authors declare that the research was conducted in the absence of any commercial or financial relationships that could be construed as a potential conflict of interest.

## Abbreviations

BLAST: Basic Local Alignment Search Tool
EDX: energy-dispersive X-ray spectroscopy
gDNA: genomic DNA
HEPES: 4-(2-hydroxyethyl)-1-piperazineethanesulfonic acid
ICP-OES: inductively coupled plasma optical emission spectrometry
LBB: leucoberbelin blue I
MCO: multicopper oxidase
Mn: manganese
MTC: maximum tolerated concentration
SEM: scanning electron microscopy
TEM: transmission electron microscopy

## References

[B1] Abo-Amer A. (2011). Biodegradation of diazinon by *Serratia marcescens* DI101 and its use in bioremediation of contaminated environment. *J. Microbiol. Biotechnol.* 21 71–80. 10.4014/jmb.1007.07024 21301195

[B2] Adams L. F. Ghiorse W. C. (1987). Characterization of extracellular Mn^2+^-oxidizing activity and isolation of an Mn^2+^-oxidizing protein from *Leptothrix discophora* SS-1. *J. Bacteriol.* 169 1279–1285. 10.1128/jb.169.3.1279-1285.1987 3818545 PMC211931

[B3] Aiyar A. (2000). The use of CLUSTAL W and CLUSTAL X for multiple sequence alignment. *Methods Mol. Biol.* 132 221–241. 10547838 10.1385/1-59259-192-2:221

[B4] Altschul S. F. Gish W. Miller W. Myers E. W. Lipman D. J. (1990). Basic local alignment search tool. *J. Mol. Biol.* 215 403–410. 10.1016/S0022-2836(05)80360-2 2231712

[B5] Bamforth S. M. Manning D. A. C. Singleton I. Younger P. L. Johnson K. L. (2006). Manganese removal from mine waters – investigating the occurrence and importance of manganese carbonates. *Appl. Geochem.* 21 1274–1287. 10.1016/j.apgeochem.2006.06.004

[B6] Barboza N. R. Amorim S. S. Santos P. A. Reis F. D. Cordeiro M. M. Guerra-Sa R. (2015). Indirect manganese removal by *Stenotrophomonas* sp. and *Lysinibacillus* sp. isolated from Brazilian mine water. *BioMed Res. Int.* 2015:925972 10.1155/2015/925972 PMC4678070 26697496

[B7] Barboza N. R. Guerra de Sa R. Leão V. A. (2016). Mechanisms of manganese bioremediation by microbes: an overview. *J. Chem. Technol. Biotechnol.* 91 2733–2739. 10.1002/jctb.4997

[B8] Beane S. J. Comber S. D. Rieuwerts J. Long P. (2016). Abandoned metal mines and their impact on receiving waters: a case study from Southwest England. *Chemosphere* 153 294–306. 10.1016/j.chemosphere.2016.03.022 27023117

[B9] Brouwers G. J. de Vrind J. P. Corstjens P. L. Cornelis P. Baysse C. de Vrind-de Jong E. W. (1999). cumA, a gene encoding a multicopper oxidase, is involved in Mn^2+^ oxidation in *Pseudomonas putida* GB-1. *Appl. Environ. Microbiol.* 65 1762–1768. 10103278 10.1128/aem.65.4.1762-1768.1999 PMC91248

[B10] Brouwers G. J. Vijgenboom E. Corstjens P. L. A. M. de Vrind J. P. M. de Vrind-de Jong E. W. (2000). Bacterial Mn^2+^ oxidizing systems and multicopper oxidases: an overview of mechanisms and functions. *Geomicrobiol. J.* 17 1–24. 10.1080/014904500270459

[B11] Burger M. S. Gagnon G. A. Mercer S. S. Shupe G. D. (2008). Manganese removal during bench-scale biofiltration. *Water Res.* 42 4733–4742. 10.1016/j.watres.2008.08.024 18809196

[B12] Chandra R. Abhishek A. Sankhwar M. (2011). Bacterial decolorization and detoxification of black liquor from rayon grade pulp manufacturing paper industry and detection of their metabolic products. *Bioresour. Technol.* 102 6429–6436. 10.1016/j.biortech.2011.03.048 21482463

[B13] Cycon M. Zmijowska A. Wojcik M. Piotrowska-Seget Z. (2013). Biodegradation and bioremediation potential of diazinon-degrading *Serratia marcescens* to remove other organophosphorus pesticides from soils. *J. Environ. Manage.* 117 7–16. 10.1016/j.jenvman.2012.12.031 23333465

[B14] Das A. P. Sukla L. B. Pradhan N. Nayak S. (2011). Manganese biomining: a review. *Bioresour. Technol.* 102 7381–7387. 10.1016/j.biortech.2011.05.018 21632238

[B15] Dick G. J. Torpey J. W. Beveridge T. J. Tebo B. M. (2008). Direct identification of a bacterial manganese(II) oxidase, the multicopper oxidase MnxG, from spores of several different marine *Bacillus* species. *Appl. Environ. Microbiol.* 74 1527–1534. 10.1128/AEM.01240-07 18165363 PMC2258647

[B16] Divekar M. (2010). *Isolation and Characterization of a Manganese Oxidizing Bacterium from the Mediterranean Marine Sponge Suberites domuncula.* Doctoral dissertation Johannes Gutenberg-Universität, Mainz.

[B17] Ehrlich H. L. (1999). Microbes as geologic agents: their role in mineral formation. *Geomicrobiol. J.* 16 135–153. 10.1080/014904599270659

[B18] Emerson D. Ghiorse W. C. (1992). Isolation, cultural maintenance, and taxonomy of a sheath-forming strain of *Leptothrix discophora* and characterization of manganese-oxidizing activity associated with the sheath. *Appl. Environ. Microbiol.* 58 4001–4010. 16348826 10.1128/aem.58.12.4001-4010.1992 PMC183217

[B19] Gallard H. van Gunten U. (2002). Chlorination of natural organic matter: kinetics of chlorination and of THM formation. *Water Res.* 36 65–74. 10.1016/S0043-1354(01)00187-7 11766819

[B20] Geszvain K. McCarthy J. K. Tebo B. M. (2013). Elimination of manganese(II,III) oxidation in *Pseudomonas putida* GB-1 by a double knockout of two putative multicopper oxidase genes. *Appl. Environ. Microbiol.* 79 k357–366. 10.1128/AEM.01850-12 PMC3536112 23124227

[B21] Hope C. K. Bott T. R. (2004). Laboratory modelling of manganese biofiltration using biofilms of *Leptothrix discophora*. *Water Res.* 38 1853–1861. 10.1016/j.watres.2003.12.031 15026240

[B22] Hullo M. F. Moszer I. Danchin A. Martin-Verstraete I. (2001). CotA of *Bacillus subtilis* is a copper-dependent laccase. *J. Bacteriol.* 183 5426–5430. 10.1128/JB.183.18.5426-5430.2001 11514528 PMC95427

[B23] Imtiaz M. Rizwan M. S. Xiong S. Li H. Ashraf M. Shahzad S. M. (2015). Vanadium, recent advancements and research prospects: a review. *Environ. Int.* 80 79–88. 10.1016/j.envint.2015.03.018 25898154

[B24] Instituto Brasileiro de Mineração [IBRAM] (2012). *Informações e análises da Economia Mineral Brasileira*, 7th Edn Brasília: Instituto Brasileiro de Mineração [IBRAM].

[B25] Johnson D. B. Hallberg K. B. (2003). The microbiology of acidic mine waters. *Res. Microbiol.* 154 466–473. 10.1016/S0923-2508(03)00114-1 14499932

[B26] Joshi B. H. Modi K. G. (2013). Screening and characterization of heavy metal resistant bacteria for its prospects in bioremediation of contaminated soil. *J. Environ. Res. Dev.* 7 1531–1538.

[B27] Klein R. Tischler J. S. Muhling M. Schlomann M. (2014). Bioremediation of mine water. *Adv. Biochem. Eng. Biotechnol.* 141 109–172. 10.1007/10_2013_265 24357145

[B28] Krumbein W. E. Altmann H. J. (1973). A new method for the detection and enumeration of manganese oxidizing and reducing microorganisms. *Helgolander Wiss. Meeresunters* 25 347–356. 10.1007/BF01611203

[B29] Kumar R. Acharya C. Joshi S. R. (2011). Isolation and analyses of uranium tolerant *Serratia marcescens* strains and their utilization for aerobic uranium U(VI) bioadsorption. *J. Microbiol.* 49 568–574. 10.1007/s12275-011-0366-0 21887639

[B30] Learman D. R. Wankel S. D. Webb S. M. Martinez N. Madden A. S. Hansel C. M. (2011). Coupled biotic–abiotic Mn(II) oxidation pathway mediates the formation and structural evolution of biogenic Mn oxides. *Geochim. Cosmochim. Acta* 75 6048–6063. 10.1016/j.gca.2011.07.026

[B31] Luan F. Santelli C. M. Hansel C. M. Burgos W. D. (2012). Defining manganese(II) removal processes in passive coal mine drainage treatment systems through laboratory incubation experiments. *Appl. Geochem.* 27 k1567–1578. 10.1016/j.apgeochem.2012.03.010

[B32] Mariner R. Johnson D. B. Hallberg K. B. (2008). Characterisation of an attenuation system for the remediation of Mn(II) contaminated waters. *Hydromettallurgy* 94 100–104. 10.1016/j.hydromet.2008.05.024

[B33] Massante J. C. (2015). Mining disaster: restore habitats now. *Nature* 528:39 10.1038/528039c 26632581

[B34] Mayhew L. E. Swanner E. D. Martin A. P. Templeton A. S. (2008). Phylogenetic relationships and functional genes: distribution of a gene (mnxG) encoding a putative manganese-oxidizing enzyme in *Bacillus* species. *Appl. Environ. Microbiol.* 74 7265–7271. 10.1128/AEM.00540-08 18849460 PMC2592937

[B35] Miyata N. Sugiyama D. Tani Y. Tsuno H. Seyama H. Sakata M. (2007). Production of biogenic manganese oxides by repeated-batch cultures of laboratory microcosms. *J. Biosci. Bioeng.* 103 432–439. 10.1263/jbb.103.432 17609158

[B36] Nealson K. H. Tebo B. M. Rosson R. A. (1988). Occurrence and mechanisms of microbial oxidation of manganese. *Adv. Appl. Microbiol.* 33 279–318. 10.1016/S0065-2164(08)70209-0

[B37] Pacini V. A. Ingallinella A. M. Sanguinetti G. (2005). Removal of iron and manganese using biological roughing up flow filtration technology. *Water Res.* 39 4463–4475. 10.1016/j.watres.2005.08.027 16225901

[B38] Peres T. V. Schettinger M. R. Chen P. Carvalho F. Avila D. S. Bowman A. B. (2016). Manganese-induced neurotoxicity: a review of its behavioral consequences and neuroprotective strategies. *BMC Pharmacol. Toxicol.* 17:57 10.1186/s40360-016-0099-0 PMC5097420 27814772

[B39] Rajasekar A. Babu T. G. Pandian S. T. Maruthamuthu S. Palaniswamy N. Rajendran A. (2007a). Role of *Serratia marcescens* ACE2 on diesel degradation and its influence on corrosion. *J. Ind. Microbiol. Biotechnol.* 34 589–598. 10.1007/s10295-007-0225-5 17605058

[B40] Rajasekar A. Babu T. G. B. Maruthamuthu S. Pandian S. T. K. Sidhan Mohanan S. M. Palaniswamy N. (2007b). Biodegradation and corrosion behaviour of *Serratia marcescens* ACE2 isolated from an Indian diesel-transporting pipeline. *World J. Microbiol.* 23 1065–1074. 10.1007/s11274-006-9332-0

[B41] Richardson L. L. Aguilar C. Nealson K. H. (1988). Manganese oxidation in pH and O_2_ microenvironments produced by phytoplankton. *Limnol. Oceanogr.* 33 352–363. 10.4319/lo.1988.33.3.0352 11538363

[B42] Saitou N. Nei M. (1987). The neighbor-joining method: a new method for reconstructing phylogenetic trees. *Mol. Biol. Evol.* 4 406–425. 3447015 10.1093/oxfordjournals.molbev.a040454

[B43] SambrooK J. Maniatis T. Fritsch E. F. (1989). *Molecular Cloning: A Laboratory Manual.* Cold Spring Harbor, NY: Cold Spring Harbor Laboratory Press.

[B44] Sethurajan M. Huguenot D. Lens P. N. Horn H. A. Figueiredo L. H. van Hullebusch E. D. (2016). Fractionation and leachability of heavy metals from aged and recent Zn metallurgical leach residues from the Tres Marias zinc plant (Minas Gerais, Brazil). *Environ. Sci. Pollut. Res.* 23 7504–7516. 10.1007/s11356-015-6014-1 26728285

[B45] Silva A. M. Cruz F. L. Lima R. M. Teixeira M. C. Leao V. A. (2010). Manganese and limestone interactions during mine water treatment. *J. Hazard. Mater.* 181 514–520. 10.1016/j.jhazmat.2010.05.044 20570440

[B46] Silva A. M. Cunha E. C. Silva F. D. R. Leão V. A. (2012). Treatment of high-manganese mine water with limestone and sodium carbonate. *J. Clean. Prod.* 29–30, 11–19. 10.1016/j.jclepro.2012.01.032

[B47] Soldatova A. V. Butterfield C. Oyerinde O. F. Tebo B. M. Spiro T. G. (2012). Multicopper oxidase involvement in both Mn(II) and Mn(III) oxidation during bacterial formation of MnO_2_. *J. Biol. Inorg. Chem.* 17 1151–1158. 10.1007/s00775-012-0928-6 22892957 PMC3743667

[B48] Su J. Bao P. Bai T. Deng L. Wu H. Liu F. (2013). CotA, a multicopper oxidase from *Bacillus pumilus* WH4, exhibits manganese-oxidase activity. *PLOS ONE* 8:e60573 10.1371/journal.pone.0060573 PMC3618234 23577125

[B49] Tang W. Gong J. Wu L. Li Y. Zhang M. Zeng X. (2016). DGGE diversity of manganese mine samples and isolation of a *Lysinibacillus* sp. efficient in removal of high Mn (II) concentrations. *Chemosphere* 165 277–283. 10.1016/j.chemosphere.2016.08.134 27657820

[B50] Tebo B. M. Bargar J. R. Clement B. G. Dick G. J. Murray K. J. Parker D. (2004). Biogenic manganese oxides: properties and mechanisms of formation. *Annu. Rev. Earth Planet. Sci.* 32 287–328. 10.1146/annurev.earth.32.101802.120213

[B51] Tebo B. M. Johnson H. A. McCarthy J. K. Templeton A. S. (2005). Geomicrobiology of manganese(II) oxidation. *Trends Microbiol.* 13 421–428. 10.1016/j.tim.2005.07.009 16054815

[B52] Tuffin I. M. Hector S. B. Deane S. M. Rawlings D. E. (2006). Resistance determinants of a highly arsenic-resistant strain of *Leptospirillum ferriphilum* isolated from a commercial biooxidation tank. *Appl. Environ. Microbiol.* 72 2247–2253. 10.1128/AEM.72.3.2247-2253.2006 16517682 PMC1393207

[B53] van Waasbergen L. G. Hildebrand M. Tebo B. (1996). Identification and characterization of a gene cluster involved in manganese oxidation by spore of marine *Bacillus* sp strain SG-1. *J. Bacteriol.* 178 3517–3530. 10.1128/jb.178.12.3517-3530.1996 8655549 PMC178121

[B54] Verma P. Madamwar D. (2003). Decolourization of synthetic dyes by a newly isolated strain of *Serratia marcescens*. *World J. Microbiol. Biotechnol.* 19 615–618. 10.1023/A:1025115801331

[B55] Wang J. Yang D. Zhang Y. Shen J. van der Gast C. Hahn M. W. (2011). Do patterns of bacterial diversity along salinity gradients differ from those observed for macroorganisms? *PLOS ONE* 6:e27597 10.1371/journal.pone.0027597 PMC3220692 22125616

[B56] Xuezheng L. Aiguo G. Haowen C. (2008). Isolation and phylogenetic analysis of cultivable manganese bacteria in sediments from The Arctic Ocean. *Acta Ecol. Sin.* 28 6364–6370. 10.1016/S1872-2032(09)60017-2

[B57] Yang W. Zhang Z. Chen H. Liu J. Ali M. Liu F. (2013). Population structure of manganese-oxidizing bacteria in stratified soils and properties of manganese oxide aggregates under manganese-complex medium enrichment. *PLOS ONE* 8:e73778 10.1371/journal.pone.0073778 PMC3772008 24069232

